# Enterobiasis as a neglected worldwide disease: a call to action

**DOI:** 10.1590/0037-8682-0290-2024

**Published:** 2024-10-28

**Authors:** Hudson Alves Pinto, Stefan Michael Geiger, Alan Lane de Melo, Vitor Luís Tenório Mati

**Affiliations:** 1 Universidade Federal de Minas Gerais, Departamento de Parasitologia, Belo Horizonte, MG, Brasil.; 2 Universidade Federal de Lavras, Departamento de Medicina, Lavras, MG, Brasil.

Dear Editor,

*Enterobius vermicularis* (Linnaeus, 1758), commonly known as pinworm, threadworm, or seatworm, is a highly prevalent and globally distributed intestinal nematode that affects people across different social classes, with recognized importance in childhood^1^ ^-^ ^3^. Factors such as institutional populations, close family contact, and poor hygiene habits create favorable conditions for its occurrence in both impoverished and developed areas worldwide^1^ ^,^ ^3^ ^-^ ^6^. A recent bibliographic survey of enterobiasis in Brazil, published in RSBMT/JBSTM (https://doi.org/10.1590/0037-8682-0073-2023)by Fantinatti and Da-Cruz^5^, highlights significant infection rates in the country, reaching up to 72.1% in children residing in orphanages, and underscores the scarcity of research available on this helminth.

However, *E. vermicularis* infections remain overlooked by health services, physicians, and researchers. A search for the term *Enterobius* on the website of this leading Latin American medical journal specializing in infectious and parasitic diseases revealed that, in the past 10 years, no study related to this nematode has been published, and surprisingly, no more than ten studies have been published in the last 50 years. This is not a regional peculiarity, as studies on *E. vermicularis* in international digital repositories from biomedical journals are scarce, and most publications deal with parasitological surveys. The number of studies found in a search of the PubMed Central database for *Ascaris lumbricoides*, another widely distributed and neglected human nematode, was at least three times greater than that observed for human pinworms. Furthermore, the omission of enterobiasis from the World Health Organization (WHO)’s list of neglected tropical diseases serves as an illustration of this situation. Recognizing the existing knowledge gaps and critically assessing the current state of *E. vermicularis* research can serve as a catalyst for advancements, particularly in infection control, as seen in Table 1.

**TABLE 1:** Facing the problem: ten topics and intriguing key questions about unresolved aspects of *Enterobius vermicularis* that can impact infection control.

Topic	Key questions
1. Basic biology	- What is the significance of autoinfection and unusual routes of transmission (e.g., retroinfection, inhalation)?
	- Do female pinworms actively migrate to the perianal region at night?
	- What is the infective stage of the larva within the nematode egg?
2. Perianal pruritus	- In addition to etiopathogenesis, what are the clinical and social consequences of perianal itching?
	- How can an integrated view improve our understanding of this symptom in enterobiasis?
3. Immune stimulation	- Does *E. vermicularis* stimulate the human immune system locally or systemically?
	- What implications does this have for infection dynamics?
4. Secretory and excretory products	- What do we know about the nematode’s secretions and excretions?
	- Could any of these antigens be harnessed for vaccine development?
5. Ectopic parasitism	- How does the nematode end up in unexpected locations in humans, and what are the associated clinical consequences?
6. Prevalence and morbidity	- Why is there a scarcity of up-to-date incidence and prevalence data worldwide?
	- What factors contribute to the lack of assessment regarding the reduced quality of life during *E. vermicularis* infection?
	- Why are years lost due to disability (YLDs), disability-adjusted life-years (DALYs), and other morbidity estimates absent for enterobiasis?
7. Diagnosis and treatment	- Why have diagnostic methods for*E. vermicularis*seen limited progress over the past century?
	- What strategies can improve diagnosis and treatment of the infection?
	- Why is there a lack of advancement in mass drug administration?
	- How to improve new drug development and treatment protocols for different populations, especially young children and pregnant or lactating women?
8. Experimental enterobiasis	- How can we develop effective models to study this infection?
9. Phylogeny and coevolution	- How does the phylogeny of*E. vermicularis*relate to current trends in coevolution research?
10. Research inertia	- How can we motivate policymakers, institutions, and researchers to overcome inertia and actively pursue studies related to enterobiasis?
	- What strategies can be implemented to enhance research efforts in this field, encompassing funding and grants, data sharing, repositories, and international cooperation?

Perhaps because enterobiasis has been traditionally considered benign, aspects of nematode biology and morbidity remain poorly understood. This lack of knowledge is more remarkable considering the effects of perianal pruritus, restlessness, irritability, and insomnia on children's psychological and cognitive development. Quantitative studies on these aspects are virtually non-existent, although they are frequently mentioned in textbooks^6^. Furthermore, evidence supporting the underestimated pathogenic potential of pinworms in humans has been highlighted based on histopathological changes, including severe inflammation, and biochemical and hematological alterations^2^ ^,^ ^4^ ^,^ ^7^ ^-^ ^9^. These findings underscore the clinical significance of the disease and raise awareness about its potential severity. The gynecological and obstetrical aspects related to the ectopic presence of pinworms or their eggs, including their potential relationship with infertility, are equally poorly discussed^10^. Moreover, the debated and controversial association between *E. vermicularis* and appendicitis has not been completely proven, but has received more evidence of a causal relationship in recent studies^8^ ^,^ ^9^. Considering these clinical uncertainties, a full assessment of the impact of infection on human health in terms of the overall disease burden, including estimates of years lost due to disability or disability-adjusted life years, remains nonexistent.

The unique biology of pinworms, especially their host specificity^1^ ^,^ ^3^ ^,^ ^6^, is likely linked to the lack of basic information about *E. vermicularis*. An alternative search of the PubMed Central database using the term ‘experimental infection’ combined with ‘*Enterobius vermicularis*’ yielded only one study, suggesting a lack of research on experimental models. Expanding the search to include broader terms such as ‘Oxyuridae’ or ‘pinworm’ resulted in just two additional experimental studies, both involving the rodent parasite *Syphacia obvelata* (Rudolphi, 1802). Advancements in our understanding of the immunology and pathophysiology of infections are crucial. For example, the mechanisms underlying the primary clinical manifestations of perianal itching have not been thoroughly studied^1^ ^,^ ^3^ ^,^ ^7^. Furthermore, aspects that have already been well researched in other human helminths, such as the characterization of secretory and excretory antigens, remain unavailable for *E. vermicularis*. Interestingly, this nematode species has only modestly entered the ‘omics’ era; there is no proteomics data, and whole-genome sequencing became available only in 2018. These studies, which include in vitro and in silico approaches, are essential for addressing the gap in basic scientific knowledge and favoring the development of new drugs, vaccines, and other control measures for enterobiasis.

The low sensitivity of routine fecal examinations for detecting pinworm eggs is also attributed to the unique characteristics of the nematode, especially the presence of female worms in the perianal region for oviposition, the mechanisms of which are not fully understood. Unfortunately, there have been no significant advancements in the parasitological diagnosis of enterobiasis since the description of methods like the ‘anal swab’ (NIH cellophane swab or Halls’ test) or ‘adhesive tape’ (Graham’s test) in the late 1930s and early 1940s^1^ ^,^ ^3^ ^,^ ^6^. Currently, personal constraints and embarrassment related to the collection of materials from the perianal region hinder the broader adoption of these methods in clinical laboratories. Thus, immunological or molecular studies, including serological tests and the detection of pinworm-specific antigens or DNA in feces, are necessary to make diagnostics more user-friendly^7^. Incidental histopathological findings and direct nematode observation can occur during colonoscopy^3^, however, this method is not routinely used for this diagnosis.

More information about treatment is still desirable as drug schedules remain largely empirical. The effectiveness of mass drug administration in *E. vermicularis* infections has not yet been evaluated, although this approach has been widely used for treatment and control of other nematodes^11^. The efficacy of benzimidazole derivatives, including mebendazole and albendazole, has been observed; however, recurrent infections and/or reinfections remain common, and there is a need for frequent, yet poorly defined, rounds of treatment^3^ ^,^ ^6^. Mebendazole exhibits reduced intestinal absorption, and is recommended as the drug of choice. However, for urogenital enterobiasis, the use of systemically effective drugs such as albendazole or ivermectin is more appropriate^3^. Addressing this challenge regarding gaps in treatment requires further clinical and epidemiological investigations, including comparisons of different drug regimens and the management of special risk groups, such as infants under six years of age and pregnant or lactating women. Additionally, in the context of basic research, utilizing non-human primates and rodents as models, even if they are infected with other species of oxyurids, could yield valuable insights that could be extrapolated to human infection.

Enhancing the awareness of the factors contributing to the delay in knowledge related to parasitosis, particularly the lack of research, is essential. Furthermore, improving the training of physicians and other health professionals, along with increased attention from health institutions and public policymakers, is necessary to address enterobiasis, a persistent health challenge despite advancements in global human helminth control^12^. Indeed, some recommended measures for the control of soil-transmitted helminths (STHs), including the causative agents of ascariasis, ancylostomiasis, strongyloidiasis, and trichuriasis^11^ ^,^ ^12^ may not affect *E. vermicularis* infection. Adequate water and sanitation-two of the three measures in the WASH approach (the other being hygiene) recommended for the prevention of STHs by the WHO-have a limited impact on pinworm infections, since the importance of domiciliary contamination and direct person-to-person transmission outweighs other routes of infection. If the WHO’s goals for STH control are successfully achieved by 2030, the impact of pinworm infection on human health may worsen owing to the future discontinuation of mass drug administration against STHs in endemic areas.

Will humans continue to coexist with *E. vermicularis* indefinitely? The answer appears to be ‘yes’ unless significant changes occur in the current situation and key questions about the parasite are thoroughly addressed and answered.
